# Anti-Inflammatory Properties of Fructo-Oligosaccharides in a Calf Lung Infection Model and in *Mannheimia haemolytica*-Infected Airway Epithelial Cells

**DOI:** 10.3390/nu13103514

**Published:** 2021-10-06

**Authors:** Yang Cai, Myrthe S. Gilbert, Walter J. J. Gerrits, Gert Folkerts, Saskia Braber

**Affiliations:** 1 Division of Pharmacology, Utrecht Institute for Pharmaceutical Sciences, Faculty of Science, Utrecht University, 3584 CG Utrecht, The Netherlands; y.cai@uu.nl (Y.C.); G.Folkerts@uu.nl (G.F.); 2 Animal Nutrition Group, Wageningen University, 6708 WD Wageningen, The Netherlands; myrthe.gilbert@wur.nl (M.S.G.); walter.gerrits@wur.nl (W.J.J.G.)

**Keywords:** respiratory diseases, pneumonia, airway inflammation, animal model, non-digestible oligosaccharides, nutrition

## Abstract

Emerging antimicrobial-resistant pathogens highlight the importance of developing novel interventions. Here, we investigated the anti-inflammatory properties of Fructo-oligosaccharides (FOS) in calf lung infections and in airway epithelial cells stimulated with pathogens, and/or bacterial components. During a natural exposure, 100 male calves were fed milk replacer with or without FOS for 8 weeks. Then, immune parameters and cytokine/chemokine levels in the bronchoalveolar lavage fluid (BALF) and blood were measured, and clinical scores were investigated. Calf primary bronchial epithelial cells (PBECs) and human airway epithelial cells (A549) were treated with *Mannheimia haemolytica*, lipopolysaccharides (LPS), and/or flagellin, with or without FOS pretreatment. Thereafter, the cytokine/chemokine levels and epithelial barrier function were examined. Relative to the control (naturally occurring lung infections), FOS-fed calves had greater macrophage numbers in BALF and lower interleukin (IL)-8, IL-6, and IL-1β concentrations in the BALF and blood. However, FOS did not affect the clinical scores. At slaughter, FOS-fed calves had a lower severity of lung lesions compared to the control. Ex vivo, FOS prevented *M. haemolytica*-induced epithelial barrier dysfunction. Moreover, FOS reduced *M. haemolytica*- and flagellin-induced (but not LPS-induced) IL-8, TNF-α, and IL-6 release in PBECs and A549 cells. Overall, FOS had anti-inflammatory properties during the natural incidence of lung infections but had no effects on clinical symptoms.

## 1. Introduction

Lung infections are a particularly common destructive public health problem in both humans and livestock, associated with high morbidity, mortality, and costs [1,2,3]. When the defense of the respiratory system is impaired, pathogens quickly invade and colonize the epithelium of the lower respiratory tract, resulting in the development of lung infections [2]. Antibiotics are the main treatment for lung infections, but the emergence of bacterial resistance necessitates more extensive research on strategies to improve (lung) health, and thus reduce the use of antibiotics.

Calves are naturally prone to respiratory infections and are demonstrated to be a valuable animal model for studying lung infections in general, due to a high natural prevalence, easy-to-observe respiratory symptoms, and the continuous production of inflammatory mediators in the airways [2]. Moreover, various bacteria and viruses can cause respiratory diseases in calves, among which *Mannheimia haemolytica* is one of the major Gram-negative bacteria which cause lung infections [2,4]. Notably, the airway epithelium forms a complex physicochemical barrier to provide the first line of defense against inhaled pathogens [5]. However, *M. haemolytica* initiates strategies to impair the airway epithelial barrier to achieve invasion and colonization, including the induction of excessive inflammatory responses [6].

Non-digestible oligosaccharides (NDOs) have the potential to reduce respiratory diseases in human and murine species due to their prebiotic and immunomodulatory properties [7,8,9]. For instance, oral pectin-derived acidic oligosaccharides (pAOS) increased the bacterial clearance and reduced the tumor necrosis factor (TNF)-α release in mice with *P. aeruginosa*-induced lung infections [8]. Moreover, the NDO mixture containing fructo-oligosaccharides (FOS) and galacto-oligosaccharides (GOS) prevented (especially respiratory) infections during the first 6 months of age [9] and reduced the frequency of respiratory infections, fever, and antibiotics in the following two years in infants [10]. In addition, different NDOs, such as human milk oligosaccharides (HMOs), FOS and GOS, can also be absorbed into the systemic circulation after oral administration [11,12,13,14] and exert direct effects on the pathogens or host cells. There is some evidence that specific NDOs, such as HMO, GOS and FOS, can directly regulate the function and signaling of Toll-like receptors (TLRs) [15,16,17], which orchestrate the inflammatory response to lung infections in both humans and calves by recognizing exogenous pathogen- or endogenous danger-associated molecular patterns [2,18]. Therefore, NDOs, such as FOS, are attractive candidates in the (direct or indirect) prevention or decline of lung infections/inflammation.

Based on these findings, we hypothesized that FOS may alleviate lung infection-induced inflammation in calves. Due to the possible presence of FOS in the systemic circulation after oral administration, we investigated whether FOS could affect the signaling of TLR4 and 5 and the subsequent inflammatory response in primary bronchial epithelial cells (PBECs), as well as whether they could restore the impaired epithelial barrier function induced by *M. haemolytica*.

## 2. Materials and Methods

### 2.1. In Vivo Experiments

#### 2.1.1. Animals and Experimental Design

This experiment was conducted under the Dutch Law on Animal Experiments in accordance with EU Directive 2010/63 at the research facilities of the VanDrie Group (Scherpenzeel, The Netherlands) and was approved by the Animal Care and Use Committee of Wageningen University (AVD1040020185828, Wageningen, The Netherlands).

One hundred male Holstein Friesian calves aged 18 days (43.2 ± 0.33 kg, means ± SEM) and of German origin were used and assigned randomly to 2 treatments. The control treatment (*n* = 50 calves) received the control milk replacer (MR). The FOS treatment (*n* = 50 calves) received 0.25% FOS (92% purity based on dry matter, DP 2–8, Frutalose OFP, Sensus, The Netherlands) in the MR at the expense of lactose (Table 1). The experiment consisted of an experimental period (week 0–8) and a growing period (week 9–27). In the experimental period (week 0–8), FOS treatment was applied, and measurements were performed. After the experimental period (week 0–8), during the growing period (week 9–27), all calves received the same MR (control MR).

During these two periods, all calves were naturally exposed to pathogens in the environment (natural incidence of disease where animals were not challenged). Individual antibiotic treatment was applied when required based on clinical signs of illness as assessed by the animal caretakers. The number of applied individual antibiotic treatments did not differ between the control and FOS groups (*p* > 0.1; 10 vs. 6 during the experimental period and 7 vs. 15 during the growing period). Group antibiotic treatment was applied equally to all groups if 10% of the calves had been treated within 5 days, or if 5% of the calves had become ill within 24 h, or when the situation required group antibiotics in the expert judgement of a veterinarian.

The in vivo study described in this article was part of a large calf trial, including 300 calves randomly assigned to a control treatment, FOS treatment, and 4 other treatments with different (dietary) interventions (50 calves/treatment). In accordance with the purpose of this study, investigating the effect of oral FOS supplementation on lung infection, we reported here the results of the analyses of the control and FOS treatments in this large calf trial.

#### 2.1.2. Housing and Diets

Calves were housed in a mechanically ventilated indoor stable throughout the experiment. The climate guidelines included that, in the first 3 weeks, a minimum temperature of 15 °C was maintained and heat canons were used when needed. Thereafter, ventilation rates were adjusted to maintain a maximum ∆T of 5 °C compared to the outside temperature and a maximum relative humidity of 80%. The ambient lighting consisted of natural lighting plus artificial lighting from 06:00 to 18:00 h. Calves were housed in pens (9 m^2^) containing wooden-slatted floors. In the first 6 weeks after arrival, individual housing was applied (1.2 m^2^/calf) by placing stainless steel fences within the pens. After 6 weeks, the individual fencing was removed, and calves were housed in groups of 5.

All calves were fed individually twice a day with MR according to a practical feeding scheme, in which MR increased progressively from 426 to 1300 g/day in the experimental period and from 1300 to 2711 g/day in the growing period. MR was mixed with warm water (66 °C) for 4 min. Cold water was added and mixed to obtain a final concentration of 125 g/L. From week 21 onwards, the concentration was gradually increased to a maximum of 181 g/L in week 27. The reconstituted MR was supplied to the calves at a temperature of approximately 42 °C. Calves were individually fed two equal MR meals at 06:00 and 16:00 h. Individual MR feeding during group housing was assured by fixing calves in the headlock of the fence. Calves were allowed access to the MR for at least 15 min. In addition, the ingredient and nutrient composition of MR diets are displayed in Table 1.

In addition to the MR, all calves received the same solid feed from 1 week after arrival and onwards. Solid feed was composed of chopped wheat straw and muesli and supplied at a ratio of 16:84 for 2 weeks and then at a ratio of 13:87. Muesli comprised corn, 412 g/kg; corn flakes, 200 g/kg; lupines, 195 g/kg; barley, 102 g/kg; molasses, 50 g/kg; vitamin-mineral mix, 25 g/kg; urea, 6 g/kg; palm oil, 5 g/kg and sodium-bicarbonate, 5 g/kg. Analyzed crude protein content was 140 g/kg dry matter. Solid feed was supplied per pen and increased progressively from 174 to 984 g/day per calf in the experimental period and from 1020 to 2620 g/day in the growing period. Feeding levels of both MR and solid feed were equal between calves.

#### 2.1.3. Sample Collection and Measurements

During the experimental period (week 0–8), clinical scores were weekly assessed for all calves (50 calves/treatment), while leukocyte parameters and cytokine/chemokine concentrations in blood and bronchoalveolar lavage fluid (BALF) were analyzed bi-weekly for a subset of calves (20 calves/treatment) unless otherwise indicated. The subset of calves included 2 calves per pen, resulting in 20 calves per treatment. Per pen, the 2 calves whose arrival body weight was closest to the mean body weight of all calves at arrival, were included in this subset. At the end of the growing period, all calves were slaughtered at week 27, and lungs were scored. The detailed number of calves per treatment and parameter were displayed in Supplementary Table S1.

##### Clinical Scores

Clinical scoring was performed weekly for all calves, according to the Wisconsin calf respiratory scoring system [19], in which a score from 0 to 3 was provided for rectal temperature, coughing, nasal discharge and behavior. Clinical score was calculated as the sum of these 4 scores.

##### Blood Sampling and Hematological Analysis

Blood samples were collected in all calves by venipuncture in the jugular vein at arrival before the first MR feeding (baseline, week 0), and then were collected repeatedly in the subset of calves at experimental weeks 2, 4 and 6. Blood samples were handled under pyrogen (i.e., endotoxin) free conditions. Blood was collected in K_2_-EDTA tubes and kept on ice for collection of plasma or kept at room temperature for analysis of leukocyte numbers the same day by fluorescence flow cytometry using a Sysmex 1800iV (Sysmex Europe GmbH, Norderstedt, Germany), respectively. Plasma was collected after centrifugation at 2000× *g* and 4 °C for 20 min and was stored at −20 °C until analysis.

##### BALF Sampling and Phenotyping

BALF samples were collected repeatedly for the same subset of calves (20 calves/treatment) at experimental weeks 1, 3, 5 and 7 and obtained by use of a technique adapted from a previous description [20]. Once the sterilized 100 cm BAL catheter was wedged in the appropriate location of the lungs, a syringe was connected to the catheter and a total of 30 mL sterile saline (37 °C) solution was infused into the tube and fluid was immediately aspirated from the bronchus. BALF (18.1 ± 0.39 mL, means ± SEM) was obtained from each calf and was stored in a 50 mL tube on ice until further analysis the same day. After transport and arrival at the lab, BALF suspension was filtered by passing through a 70 μm cell strainer (Corning, New York, NY, USA) to remove debris. Then, BALF suspension was centrifuged (5 min, 400× *g* at 4 °C) and the remaining cell pellet was re-suspended in 1 mL cold fetal bovine serum (FBS). After centrifugation, the supernatant was aliquoted into 1.5 mL tubes and stored at −80 °C for further analysis. The number of the remaining cell pellet was determined by automatically counting in Cellometer Bright Field cell counter (Nexcelom Bioscience, Lawrence, MA, USA). After counting, 0.5 × 10^6^ cells of BALF suspension were used to make cytospins and phenotyping was determined by Diff-Quick (Medion Diagnostics, Medion Diagnostics International Inc., Miami, FL, USA) staining on cytospin preparations and a minimum of 400 cells were counted.

##### Measurements of Cytokines/Chemokines and *M. haemolytica*-LPS lgG

BALF and blood were collected and stored as described above. Concentrations of IL-8 (Mabtech, Nacka Strand, Sweden), IL-6 (Invitrogen, ThermoFisher Scientific, Waltham, MA, USA), IL-1β (Invitrogen) and TNF-α (R&D Systems, Minneapolis, MN, USA) in BALF and blood were determined by using ELISA kits according to manufacturer’s instructions. *M. haemolytica*-LPS lgG levels were measured to determine the *M. haemolytica* positivity in BALF according to manufacturer’s instructions (BIO/K-139, Bio-X Diagnostics, Rochefort, Belgium).

##### Lung Scores

Lungs of all remaining calves were scored at slaughter according to a scoring system adapted from Leruste et al. [21]. Briefly, each examined lung was classified according to a 4-point scale for pneumonia from healthy lung (score 0) to severe lesions (score 3). Score 0 was given for healthy lungs (pale orange color with no sign of pneumonia), score 1 for minimal or mild lesions (one spot of grey-red discoloration), score 2 for moderate lesions (one large or several small spots of grey-red discoloration with a total surface of less than 1 lobe), and score 3 for severe lesions (grey-red discoloration area of at least one full lobe and/or presence of abscesses). The results are shown as a percentage of total calves with different severity of lung lesions, including no/mild and moderate/severe lesions.

### 2.2. In Vitro Experiments

#### 2.2.1. *M. haemolytica* Growth, Quantification and Preparation

*M. haemolytica* (isolated from a pneumonic bovine lung) was kindly provided by Prof. Jos van Putten (Utrecht University, Utrecht, The Netherlands). *M. haemolytica* was incubated overnight at 37 °C in 5% sheep blood agar (bioTRADING, Mijdrecht, The Netherlands).

Subsequently, *M. haemolytica* (1 × 10^5^ CFU/mL) was incubated in 96-well plates with or without FOS (0.125%, 0.25%, 0.5%, 1%, or 2%; same FOS as in vivo experiment) for 24 h. After incubation, *M. haemolytica* growth was determined by measuring the turbidity of supernatants at OD600 nm using a microplate reader (Promega Corp., Madison, WI, USA). Then, the supernatants were diluted 50,000-fold and sub-cultured onto 5% sheep blood agar plates overnight at 37 °C. *M. haemolytica* numbers were determined by counting CFUs of each plate.

*M. haemolytica* (1 × 10^5^ CFU/mL) were cultured in RPMI-1640 medium containing 10% FBS (Sigma-Aldrich, Zwijndrecht, The Netherlands) for 24 h on 6-well plates. Thereafter, the *M. haemolytica*-cultured supernatants (MHS) were collected and filtered with a 0.25 μm filter (GE Healthcare, Chicago, IL, USA).

#### 2.2.2. Cell Cultures and Treatments

Isolation and culture of PBECs were conducted as previously described [6]. Briefly, PBECs were isolated from bovine bronchial epithelium obtained from the lungs of freshly slaughtered calves aged 6–8 months, provided by Ekro bv (Apeldoorn, The Netherlands). After digesting of the bronchial epithelium, PBECs were collected and grown in 5% CO_2_ at 37 °C and attached to collagen-coated plates in serum-free RPMI-1640 medium for 2–3 days until reaching near-confluence, and then replaced with RPMI-1640 medium containing 10% FBS, 1% L-glutamine, 1% MEM NEAA, and 1% penicillin–streptomycin (Sigma-Aldrich). After that, these PBECs (1 × 10^6^ cells/mL) were first pretreated with different concentrations of FOS (0.125%, 0.25%, or 0.5%) for 24 h and then were stimulated with LPS (10 µg/mL; isolated from *E. coli* O111:B4, Sigma-Aldrich), flagellin (10 ng/mL; isolated from *P. aeruginosa*, InvivoGen, San Diego, CA, USA), *M. haemolytica* (1 × 10^5^ CFU/mL) or MHS for 24 h. After stimulation, supernatants were collected and stored at −20 °C until analysis.

Human Type II alveolar epithelial cells (A549; ATCC, Manassas, VA, USA) were grown in Ham’s F-12K Medium (Gibco, Thermo Fisher Scientific, Waltham, MA, USA) supplemented with 10% FBS and 1% penicillin–streptomycin (Sigma-Aldrich) in 5% CO_2_ at 37 °C. After reaching near-confluence, A549 cells (0.5 × 10^5^ cells/mL) were pretreated with 0.5% FOS for 24 h prior to 24 h LPS (10 μg/mL; isolated from *E. coli* O111:B4, Sigma-Aldrich) or flagellin (10 ng/mL; isolated from *P. aeruginosa*, InvivoGen) stimulation. After stimulation, supernatants were collected and stored at −20 °C until analysis.

#### 2.2.3. Transepithelial Electrical Resistance (TEER) Measurement and Paracellular Tracer Flux Assay

PBECs (1 × 10^6^ cells/mL, 300 µL) were added to the apical compartment of the permeable 0.3 cm^2^ high pore density polyethylene membrane transwell inserts (Corning) placed in a 24-well plate and 700 µL cell culture medium was added to the basolateral compartment. After that, PBECs were incubated at 37 °C in 5% CO_2_. TEER of PBECs was measured by a Millicell-ERS Volt-Ohm meter (Millipore, Merck, Darmstadt, Germany) every 2 days. The culture medium from the basolateral and apical compartment was refreshed after TEER measurement and experiments started at day 11 when sustained TEER values around 600 Ω·cm^2^ were reached as described before [6].

PBECs were incubated with *M. haemolytica* (1 × 10^5^ CFU/mL) from the apical side with or without 24 h pretreatment with FOS at both apical and basolateral compartments. TEER was measured at 0 and 24 h after *M. haemolytica* exposure. Thereafter, a membrane-impermeable molecule, lucifer yellow (molecular mass of 0.457 kDa, 20 μg/mL; Sigma-Aldrich), was added to the apical compartment for 4 h, and the paracellular flux was determined by measuring the fluorescence intensity at the basolateral compartment with a fluorometer (Promega Corp.) at excitation/emission wavelengths of 410/520 nm. After measuring, PBECs were harvested for immunofluorescence staining and Western blotting.

#### 2.2.4. ELISA Measurement and *Lactate dehydrogenase* (LDH) Assay

Levels of IL-8 (Mabtech), IL-6 (Invitrogen), IL-1β (Invitrogen) and TNF-α (R&D Systems) were determined in the supernatants of PBECs after different treatments by using ELISA kits according to manufacturer’s instructions. IL-8 (R&D Systems) and IL-6 (BioLegend, San Diego, CA, USA) release in the supernatants of A549 after different treatments were also measured by ELISA. The absorbance was measured at 450 nm using a microplate reader (Bio-Rad, Hercules, CA, USA).

LDH was measured in the supernatants of PBECs or A549 cells after different treatments using the CytoTox 96 nonradioactive cytotoxicity assay kit (Promega Corp.) according to manufacturer’s instructions [6].

#### 2.2.5. Western Blotting

Western blotting of cell lysates in transwells after different treatments and the information of primary/secondary antibodies were described previously [6]. Digital images were acquired with the Molecular Imager (Gel DocTM XR, Bio-Rad) and analyzed with Image lab 5.0 (Bio-Rad).

#### 2.2.6. Immunofluorescence

PBECs were grown in transwells as described above and detected for the tight junction protein zonula occludens-1 (ZO-1) and the adherens junction protein, E-cadherin, using immunofluorescence as previously described [6]. ZO-1 and E-cadherin were visualized, and images were taken using the Keyence BZ-9000 (Osaka, Japan). Fluorescence intensity was quantified by Image J (Version 1.8.0, National Institutes of Health, Bethesda, MD, USA) and presented as fluorescence intensity (vs. DAPI).

### 2.3. Statistical Analysis

Experimental in vivo results were expressed as non-transformed means ± standard error of mean (SEM). Blood leukocyte and cytokine/chemokine concentrations, BALF total cell, macrophage, neutrophil, and lymphocyte numbers and BALF cytokine/chemokine concentrations were analyzed for treatment and/or time effects with SAS 9.4 (SAS Institute Inc., Cary, NC, USA), using the MIXED procedure, including time as a repeated statement with calf as unit. For each parameter, the covariance structure was selected based on the lowest Akaike Information Criterion (AIC) and Bayesian Information Criterion (BIC). All analyses included a random pen effect. For blood leukocyte concentration, the concentration at arrival (before application of the treatments) was included as a co-variable in the model. Studentized residuals of each model were checked visually on the homogeneity of variance and data were transformed if required to obtain homogeneity of variance. To evaluate differences between treatments, the contrast statement was used, and treatment differences were assessed per timepoint separately. Clinical scores were assessed for treatment and time effects using the GLIMMIX procedure with a multinomial distribution, including a random pen effect and potential differences between the treatments, which were evaluated using the contrast statement per time point. The Chi-square test was performed for the proportion of different lung lesions and the positivity of *M. haemolytica* in calves. Differences were considered significant when *p* < 0.05 and considered a trend when *p* < 0.10.

Data from in vitro experiments were first checked for normality in the use of the Shapiro–Wilk normality test. After that, the parametric one-way ANOVA or two-way ANOVA with Tukey’s post hoc test was performed for the analyses of cytokine/chemokine release, TEER value, lucifer yellow flux, protein and fluorescence expression, and *M. haemolytica* growth/CFUs and LDH release. All in vitro results were expressed as means ± SEM and analyzed using the GraphPad Prism version 7.0 software (GraphPad Software Inc., San Diego, CA, USA). Results were considered significant when *p* < 0.05.

## 3. Results

### 3.1. Effect of FOS on BALF and Blood Cell Compositions and Clinical and Lung Scores in Calves

**Figure 1:**
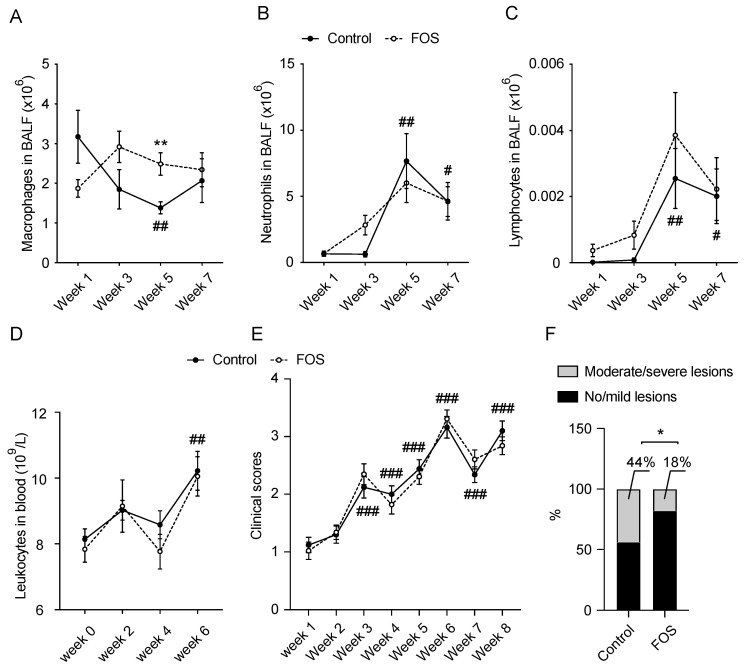
Effect of FOS on BALF and blood cell compositions and clinical and lung scores in calves. Numbers of (**A**) macrophages, (**B**) neutrophils, and (**C**) lymphocytes in BALF and (**D**) leukocyte concentrations in blood were analyzed for a subset of calves (*n* = 40, 20 calves/treatment). (**E**) Clinical scores and (**F**) proportion of different lung lesions at slaughter were evaluated for all calves (*n* = 100, 50 calves/treatment). Significantly different from week 0 or 1 for control: # *p* < 0.05; ## *p* < 0.01; ### *p* < 0.001. Significantly different from control at that time: * *p* < 0.05; ** *p* < 0.01. Data are presented as means ± SEM unless otherwise indicated. Data are presented as percentage for panel F. BALF, bronchoalveolar lavage fluid; FOS, fructo-oligosaccharides.

Lung infections were displayed in the naturally exposed calves from week 5 (Figure 1A–E and Supplementary Figure S1). A decreased number of macrophages (*p* < 0.01) and increased number of neutrophils (*p* < 0.01) and lymphocytes (*p* < 0.01) in BALF were observed from week 5 in the control calves, compared to week 1 (Figure 1A–C). The potentially increased total cells (mainly pulmonary leukocytes) in BALF (*p* = 0.09) and increased leukocytes in blood (*p* < 0.01) of control calves were observed at week 5 and 6, compared to week 1 and 0, respectively (Supplementary Figure S1 and Figure 1D). In addition, the clinical scores increased significantly over time with the peak at week 6 (*p* < 0.001), indicating the presence of lung infections (Figure 1E).

FOS-fed calves showed a greater number of BALF macrophages at week 5 (*p* < 0.01), but no effects on the other parameters of the BALF and blood cell composition were observed (Figure 1A–D and Supplementary Figure S1). The calves fed with dietary FOS showed a lower proportion of moderate/severe lung lesions (18%, *p* < 0.05) compared with the control calves (44%, Figure 1F) at slaughter (week 27). However, FOS had no effects on body weight (data not shown) and clinical scores (Figure 1E) during the experimental period.

*M. haemolytica* positivity was investigated in BALF over time. Compared with week 1 (0%), the number of *M. haemolytica* positive calves in the control treatment increased to 80% at week 5 (*p* < 0.001) and 80% at week 7 (*p* < 0.001). A proportion of *M. haemolytica*-positive calves was unaffected by dietary FOS (Supplementary Table S2).

### 3.2. Effect of FOS on Cytokine/Chemokine Concentrations in BALF and Blood of Calves

**Figure 2:**
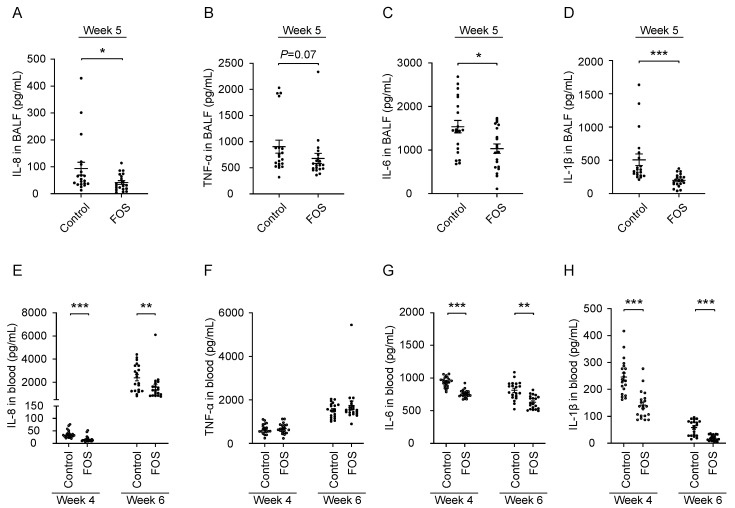
Effect of FOS on cytokine/chemokine concentrations in BALF and blood of calves. (**A** and **E**) IL-8, (**B** and **F**) TNF-α, (**C** and **G**) IL-6, and (**D** and **H**) IL-1β concentrations in BALF and blood were measured for a subset of calves (*n* = 40, 20 calves/treatment). Significantly different from control at that time: * *p* < 0.05; ** *p* < 0.01; *** *p* < 0.001. Data are presented as means ± SEM. Each dot represents one calf. BALF, bronchoalveolar lavage fluid; FOS, fructo-oligosaccharides; IL, interleukin; TNF, tumor necrosis factor.

The inflammatory response in the lungs was investigated by measuring the cytokines/chemokines in the BALF at week 5. FOS lowered the concentrations of IL-8 (*p* < 0.05), IL-6 (*p* < 0.05), and IL-1β (*p* < 0.001), and tended to also lower the concentration of TNF-α (*p* = 0.07) in BALF at week 5 (Figure 2A–D). The same cytokines/chemokines were measured in blood to investigate the effect of FOS on the systemic inflammation at weeks 4 and 6. Oral FOS lowered the concentrations of IL-8 (*p* < 0.001 and *p* < 0.01, respectively), IL-6 (*p* < 0.001 and *p* < 0.01, respectively) and IL-1β (*p* < 0.001 and *p* < 0.001, respectively) in blood at both week 4 and week 6, while no effects on the TNF-α levels were observed (Figure 2E–H).

### 3.3. Effect of FOS on M. haemolytica Growth In Vitro

**Figure 3:**
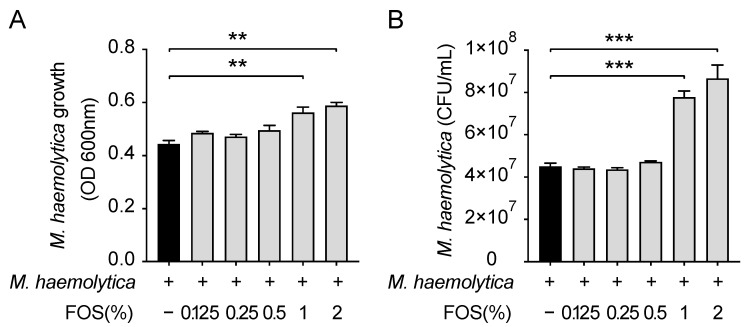
Effect of FOS on *M. haemolytica* growth in vitro. (**A**) *M. haemolytica* growth (OD600 nm) in the supernatant and (**B**) *M. haemolytica* numbers (CFUs) after subculture of supernatant on the sheep blood agar. Significantly different from *M. haemolytica* without FOS: ** *p* < 0.01; *** *p* < 0.001. Data are presented as means ± SEM. All data shown are representative of five independent experiments (*n* = 5 bacterial generations, one generation per experiment). CFU, colony-forming unit; FOS, fructo-oligosaccharides.

Due to the possible presence of FOS in the systemic circulation after oral administration [11,12,13,14], different NDOs may exert direct effects on the airway pathogens or epithelial cells. Here, 1% and 2% FOS significantly increased the growth (*p* < 0.01 and *p* < 0.01, respectively) and CFUs (*p* < 0.001 and *p* < 0.001, respectively) of *M. haemolytica* in vitro, while FOS < 1% did not (Figure 3).

### 3.4. Effect of FOS on M. haemolytica-Induced Inflammation in PBECs

**Figure 4:**
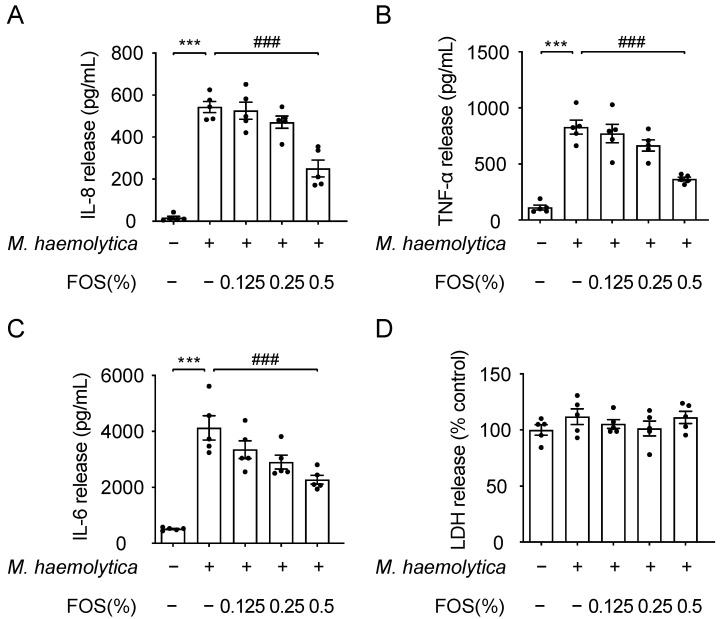
Effect of FOS on *M. haemolytica*-induced inflammation in PBECs. (**A**) IL-8, (**B**) TNF-α, (**C**) IL-6, and (**D**) LDH release were assessed in the supernatants of PBECs. Significantly different from control: *** *p* < 0.001. Significantly different from *M. haemolytica*: ### *p* < 0.001. Data are presented as means ± SEM. All data shown are representative of five independent experiments (*n* = 5 donor calves, one donor calf per experiment). FOS, fructo-oligosaccharides; IL, interleukin; LDH, lactate dehydrogenase; TNF, tumor necrosis factor.

Anti-inflammatory effects of FOS were investigated in an ex vivo infection model based on PBECs of healthy calf lungs [6]. *M. haemolytica* induced the release of IL-8 (*p* < 0.001), TNF-α (*p* < 0.001) and IL-6 (*p* < 0.001) in PBECs, while the release was significantly inhibited by the pretreatment with 0.5% FOS (*p* < 0.001, *p* < 0.001, and *p* < 0.001, respectively) (Figure 4A–C). Moreover, *M. haemolytica* with or without the pre-administration of FOS did not affect LDH release in PBECs (Figure 4D).

MHS were used as a stimulant for PBECs to investigate whether virulence factors might play a role in the observed anti-inflammatory effect of FOS. MHS successfully induced the release of IL-8 (*p* < 0.001), TNF-α (*p* < 0.001), and IL-6 (*p* < 0.001) in PBECs, while it did not affect the LDH release (Supplementary Figure S2). Pre-incubation with 0.5% FOS inhibited the MHS-induced IL-8 (*p* < 0.001), TNF-α (*p* < 0.01), and IL-6 (*p* < 0.01) release in PBECs, while no effects in the treatment with FOS alone were observed (Supplementary Figure S2).

### 3.5. Effect of FOS on M. haemolytica-Induced Barrier Dysfunction in PBECs

**Figure 5:**
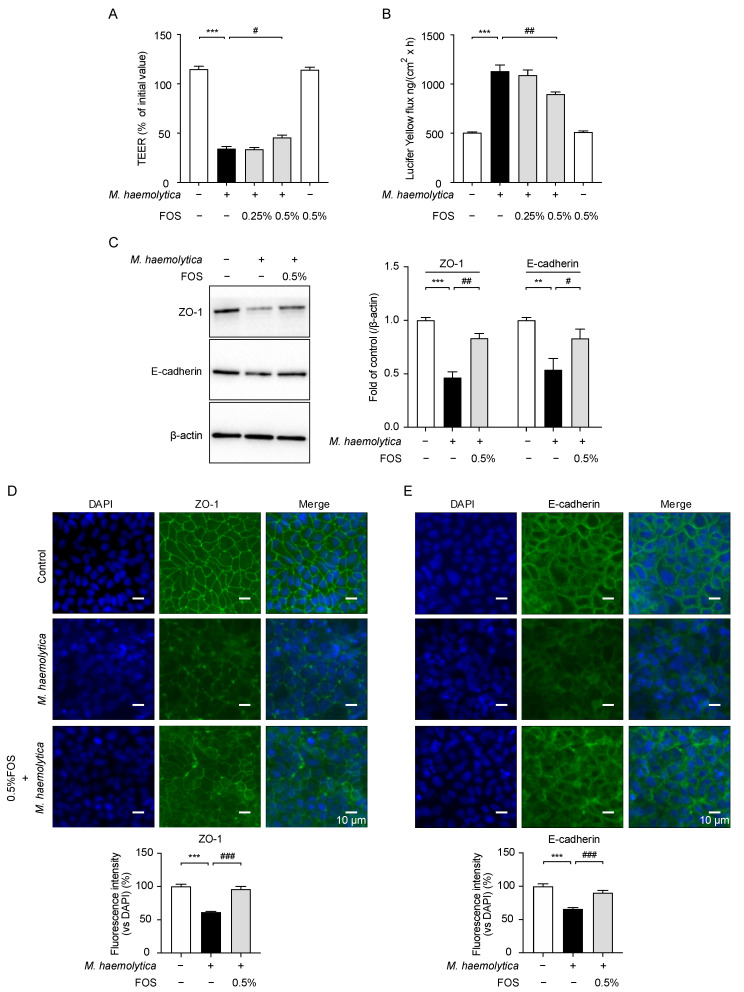
Effect of FOS on *M. haemolytica*-induced barrier dysfunction in PBECs. (**A**) TEER was measured and (**B**) lucifer yellow flux from apical to basolateral compartment was determined in PBECs. (**C**) The immunoblots were obtained with ZO-1, E-cadherin, and β-actin (protein loading control). (**D**,**E**) Cellular expression of ZO-1 and E-cadherin in PBECs was assessed by immunofluorescent staining and quantified as a percentage of fluorescence intensity. Significantly different from control: ** *p* < 0.01; *** *p* < 0.001. Significantly different from *M. haemolytica*: # *p* < 0.05; ## *p* < 0.01; ### *p* < 0.001. Data are presented as means ± SEM. All data shown are representative of at least five independent experiments (*n* = 5–6 donor calves, one donor calf per experiment). DAPI, 4′, 6-diamidino-2-phenylindole; FOS, fructo-oligosaccharides; TEER, transepithelial electrical resistance; ZO-1, zonula occludens-1.

Another characteristic of *M. haemolytica*-treated airway epithelial cells was the disruption of the epithelial barrier function [6,22]. *M. haemolytica* exposure resulted in a significant decrease in TEER (*p* < 0.001; Figure 5A), which facilitated the translocation of lucifer yellow from the apical to the basolateral compartment (*p* < 0.001; Figure 5B). A reduction in the protein level and distribution of the tight junction protein ZO-1 (*p* < 0.001 and *p* < 0.001, respectively) and the adherens junction protein E-cadherin (*p* < 0.01 and *p* < 0.001, respectively) was also observed in *M. haemolytica*-treated PBECs (Figure 5C–E). The pretreatment with 0.5% FOS prevented the *M. haemolytica*-induced TEER decrease (*p* < 0.05; Figure 5A) and increase in lucifer yellow flux (*p* < 0.01; Figure 5B), as well as the reduction in ZO-1 (*p* < 0.01 and *p* < 0.001, respectively) and E-cadherin (*p* < 0.05 and *p* < 0.001, respectively) expression (Figure 5C–E).

### 3.6. Effect of FOS on M. haemolytica-Induced Phosphorylation of Mitogen-Activated Protein Kinase (MAPK) and Nuclear Factor (NF)-κB p65 in PBECs

**Figure 6:**
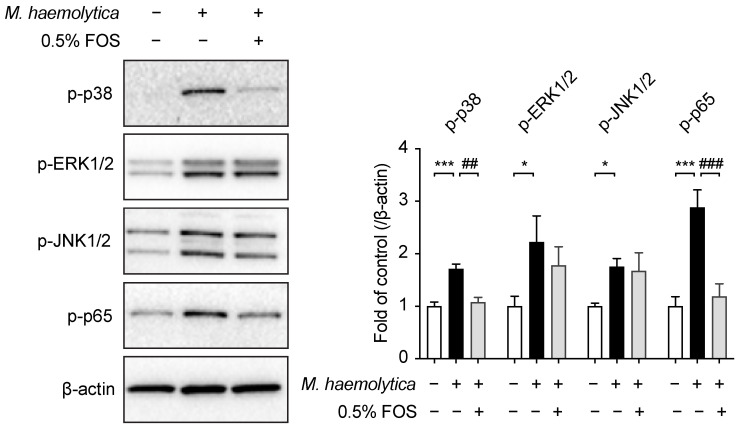
Effect of FOS on *M. haemolytica*-induced phosphorylation of MAPK and NF-κB p65 in PBECs. Phosphorylation of p38, ERK1/2, JNK1/2 MAPK and NF-κB p65 were determined by immunoblot and results were shown as a fold of control. Significantly different from control: * *p* < 0.05; *** *p* < 0.001. Significantly different from *M. haemolytica*: ## *p* < 0.01; ### *p* < 0.001. Data are presented as means ± SEM. All data shown are representative of five independent experiments (*n* = five donor calves, one donor calf per experiment). ERK, extracellular signal-regulated kinase; FOS, fructo-oligosaccharides; JNK, c-Jun N-terminal kinase; MAPK, mitogen-activated protein kinase; NF-κB, nuclear factor-κB.

The activation of the MAPK and NF-κB signaling pathways played an important role in the *M. haemolytica*-induced inflammation and barrier dysfunction in PBECs, as observed in our previous study [6]. The pretreatment with 0.5% FOS showed a decrease in the phosphorylation of p38 MAPK (*p* < 0.01) and NF-κB p65 (*p* < 0.001) in PBECs after *M. haemolytica* exposure (Figure 6). However, FOS did not affect the phosphorylation of extracellular signal-regulated kinase (ERK)1/2 and c-Jun N-terminal kinase (JNK)1/2 MAPK in the *M. haemolytica*-treated PBECs (Figure 6).

### 3.7. The Anti-Inflammatory Effect of FOS Might Be Related to the Interference with TLR5

**Figure 7:**
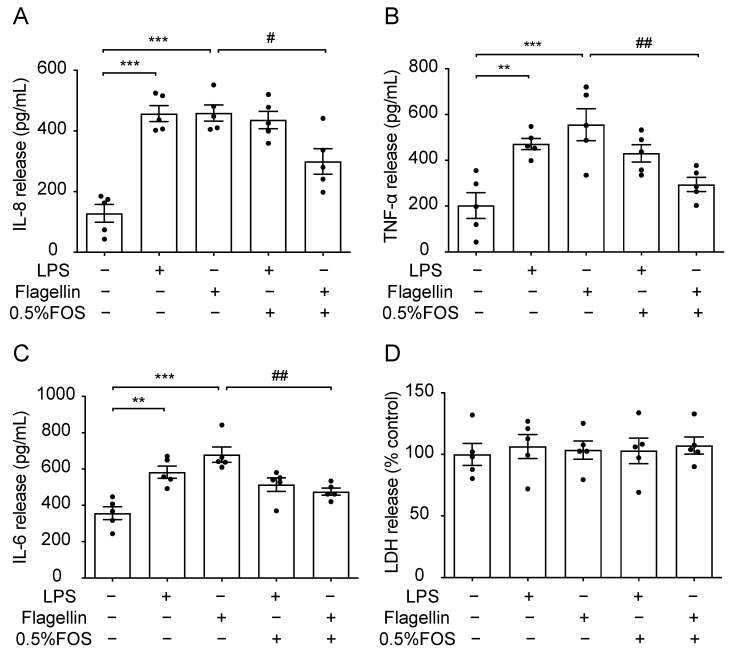
Anti-inflammatory effects of FOS in LPS- or flagellin-treated PBECs. (**A**) IL-8, (**B**) TNF-α, (**C**) IL-6, and (**D**) LDH release were determined in the supernatants of LPS- or flagellin-treated PBECs. Significantly different from control: ** *p* < 0.01; *** *p* < 0.001. Significantly different from flagellin: # *p* < 0.05; ## *p* < 0.01. Data are presented as means ± SEM. All data shown are representative of five independent experiments (*n* = 5 donor calves, one donor calf per experiment). FOS, fructo-oligosaccharides; IL, interleukin; LDH, lactate dehydrogenase; LPS, lipopolysaccharides; TNF, tumor necrosis factor.

Recent studies suggested that NDOs could exert anti-inflammatory effects via TLRs (e.g., TLR4 and TLR5) in host cells [17,23]. FOS suppressed the flagellin (TLR5 ligand)- but not the LPS (TLR4 ligand)-induced release of IL-8 (*p* < 0.05), TNF-α (*p* < 0.01), and IL-6 (*p* < 0.01) (Figure 7A–C) in PBECs, while it did not affect the LDH release (Figure 7D). In a human alveolar basal epithelial cell line (A549), FOS inhibited the flagellin-induced release of IL-8 (*p* < 0.001) and TNF-α (*p* < 0.05), but not IL-6 (Supplementary Figure S3D–F). FOS did not affect the LPS-induced cytokine/chemokine release and corresponding LDH release in A549 cells (Supplementary Figure S3A–C, G).

## 4. Discussion

This study investigated whether FOS could reduce lung infection and subsequent inflammation. Here, calves naturally exposed to environmental pathogens and *M. haemolytica*-stimulated PBECs were used to investigate the anti-inflammatory properties of FOS. Different studies demonstrated that NDOs (e.g., FOS, GOS) had beneficial effects on organs besides the intestine, including the airways [7,24], and were therefore a suitable candidate to investigate early dietary intervention to reduce lung infections.

The calf is considered to be a suitable animal model for studying lung infections: the prevalence of pneumonia is extremely high, due to airborne and close contact transmission [2]. However, during natural exposure (in the current study) or in inoculated lung infection models, clinically healthy or asymptomatic individuals were always present [25,26]. In order to prevent a high mortality, antibiotic strategies were applied for all calves in the present study, which could postpone the occurrence of lung infections and reduce the severity of lung infections. This could be the reason why the effect of oral FOS on BALF cell composition and clinical scores might be underestimated.

Eighty percent of the control calves at week 5 were positive for *M. haemolytica* (in BALF). We observed that FOS could inhibit the *M. haemolytica*-induced inflammation in PBECs (ex vivo), and there were indications that FOS could be absorbed into the systemic circulation after oral administration [11,12,13,14]. Therefore, it might be possible that FOS specifically inhibited the *M. haemolytica*-induced inflammation in calves. The insensitivity of clinical scores to diagnose (subclinical) lung infections may also lead to contrasting results as compared to the measurements of cell composition and cytokine/chemokine levels in the BALF/blood [27]. Although the clinical scores did not differ, the lung lesions at slaughter appeared less severe for FOS-supplemented calves. In the future, additional experiments with different dosages, different treatment periods, and different routes of application are needed to optimize FOS supplementation in clinical practice.

FOS intervention was applied during the first 8 weeks after arriving at the facility, since calves were clearly at the highest risk of naturally occurring lung infections within the first 8 weeks after transport/arrival [28] and, during these first two months after transport/arrival, the calves also received frequent antibacterial drugs to treat such (lung) infections. The calves in the present study originated from a diverse background (i.e., different farms), were transported under stressful conditions, had to deal with dietary changes, and were therefore at a particularly high risk of respiratory diseases. Although the calves did not receive FOS intervention after experimental week 8, the FOS-fed calves still showed a reduced severity of lung lesions after 27 weeks, which might be due to the reduction in inflammatory infiltration caused by the decreased cytokine/chemokine levels. In addition, elevated macrophage numbers in the BALF induced by oral FOS might contribute to reducing lung lesions and inflammation [2]. Most reported studies focused on the well-known intestinal protection and prebiotic effects of oligosaccharides, while there were few studies related to respiratory diseases. For instance, a dietary intervention with 1% GOS suppressed the increased BALF leukocyte numbers and CCL5 and IL-13 levels in a murine house dust mite-induced asthma model [29]. Additionally, a mixture of GOS/lcFOS/pectin supplementation decreased the BALF neutrophil numbers in mice with LPS-induced lung emphysema [7]. Moreover, dietary pAOS recruited polynuclear leukocytes and macrophages, and decreased TNF-α release in the lung, leading to an increased bacterial clearance after *P. aeruginosa* infection in mice [8]. In the present study, dietary FOS increased the total cells (mainly macrophages) and macrophages in the BALF at weeks 3 and 5, respectively. In particular, the increased macrophages might be helpful in contributing to bacterial clearance after lung infections in calves. It was reported that alveolar macrophages, as resident immune cells in the epithelial mucosa of the lower respiratory tract, played an important role in respiratory defense, including the rapid response to phagocytose pathogens and cell debris [2].

The promising anti-inflammatory effects of FOS both systemically and locally, as observed by the lower IL-8, IL-6, and IL-1β levels in the BALF and blood, might be (partly) related to the crosstalk between the gut (rumen) microbiota and the lungs, i.e., the gut–lung axis [30]. In a randomized trial with children, consuming long-chain FOS (lcFOS) resulted in a higher relative abundance of *Bifidobacteria* and *Lactobacillus* in feces and fewer incidence of febrile episodes and sinusitis [31]. An in vitro study found that FOS fermentation resulted in greater acetate and butyrate concentrations and enhanced growth of *Lactobacilli*, *Streptococci* and lactic acid utilizing bacteria in veal calf ileal contents [32]. In the present study, more dietary fibers (than FOS) were present in the MR (e.g., pea fiber) and solid feed (e.g., wheat straw and barley) of all calves. Most likely, the amount of short-chain fatty acids (SCFAs) produced from those additional fiber sources was much higher than compared to the SCFA related the FOS supplementation.

In addition to the possible indirect mechanisms via the gut–lung axis and inhibited systemic inflammation, there might be a possibility that FOS directly interacts with airway epithelial cells and respiratory pathogens after oral administration. There is some evidence that FOS have the ability to cross the intestinal epithelial barrier and might reach the lungs (bronchus) through blood circulation [11,12,13,14], resulting in the direct inhibition of inflammation and protection of the epithelial barrier function in the airways. In an experiment in rats, different HMOs (2′-fucosyllactose, 2′-FL; 6’-sialyllactose, 6′-SL; and lacto-N-neotetraose, LNnT) administered orally were detected in blood and urine [11]. Our previous study with piglets reported that, after the oral ingestion of GOS (0.8% GOS, DP 2–6, once per day), around 0.1% GOS was observed in blood serum and approximately 0.85 g GOS/g creatinine was found in urine [13]. Moreover, Eiwegger et al. showed in vitro evidence for the transport of prebiotic oligosaccharides, similar to FOS, across the intestinal epithelial layer, whereas the lower DPs of the oligosaccharides exerted a higher transmission in crossing the intestinal epithelial layer [14]. In the present study, FOS (DP 2–8) supplemented to MR might cross the intestinal epithelial layer and exert direct (systemic) effects on other organs, such as the lungs. However, we did not investigate the in vivo fate of FOS in the calves used in this study; therefore, the exact concentration of FOS in local lung tissue and systemic circulation of calves/ruminants is not available and warrants further research.

Previously studies often focused on the beneficial effects of FOS on intestinal cells, while here we investigated the direct effects of FOS on the airway epithelial cells. 0.5% FOS inhibited the release of cytokines/chemokines (IL-8, IL-6, and TNF-α) in *M. haemolytica*-stimulated PBECs, indeed suggesting the possible direct anti-inflammatory capacity of FOS in the airways. Concentrations higher than 0.5% FOS were not investigated in this in vitro study as > 0.5% FOS promoted the growth of *M. haemolytica*. It can be suggested that FOS might be used as a beneficial carbon source for *M. haemolytica* growth and survival in vitro. Additionally, we can exclude the possibility that the anti-inflammatory effects of FOS observed in vitro are related to *M. haemolytica* growth inhibition or bacterial killing. The accumulation of inflammatory cytokines (e.g., IL-6 and TNF-α) caused by infections in the airways is one of the most significant causes for the sustained impairment of the epithelial barrier function [2,33,34,35]. We showed that FOS have the ability to inhibit the levels of these cytokines in vitro and in vivo, thereby protecting the integrity of the epithelial barrier, as observed by a higher TEER value and the expression of tight/adherens junction proteins, and the lower lucifer yellow flux in PBECs. In addition, FOS may have direct protective effects on the epithelial barrier via the modulation of host cell signaling in the epithelium [36]. Our previous study indicated that 2% FOS could significantly modulate the deoxynivalenol-induced epithelial barrier disruption in Caco-2 cells [37].

In studies describing the anti-inflammatory properties of NDOs, the potential role of TLR modulation, such as TLR4 and 5 regulations, in cytokine production has been demonstrated [15,23]. It has also been reported that specific NDOs, such as FOS and GOS, may affect the inflammatory signal transduction pathways mediated by TLRs/NF-κB and secondarily by MAPK [16]. In the present study, FOS were able to inhibit the *M. haemolytica*-induced release of cytokines/chemokines (IL-8, IL-6, and TNF-α) and the phosphorylation of NF-κB p65 and p38 MAPK in PBECs. Our studies with MHS-stimulated PBECs showed that the anti-inflammatory effects of FOS were not related to the direct interaction with *M. haemolytica*, but other factors, such as virulence factors and other mediators may play a role in this process. We observed that FOS inhibited flagellin- but not LPS-induced inflammatory responses, which might be related to the interference with the TLR5 signaling in PBECs and A549 cells. Specific HMOs (2′-FL, 6′-SL, LNnT, and 3-fucosyllactose) also suppressed the flagellin-induced TLR5 activation in HEK-Blue hTLR5 cells but did not affect the LPS-induced TLR4 activation in HEK-Blue hTLR4 cells [23]. There is no evidence in the literature that *M. haemolytica* produces flagellin; therefore, FOS might inhibit the inflammatory response caused by other virulence factors (e.g., LPS) by interfering with TLR5 signaling. It could be possible that higher FOS concentrations are needed to modulate the LPS-induced inflammatory response in PBECs. A recent study in TLR5-deficient mice showed that a decreased cytokine expression in the lungs was observed after LPS exposure in the lungs, which might be due to the participation of TLR5 in TLR4-dependent signaling [38].

## 5. Conclusions

Overall, to our knowledge, this is the first study that used FOS to relieve lung infections in calves. Interestingly, FOS had the capability to reduce lung inflammation in infected calves, systemically and locally, and prevent *M. haemolytica*-induced epithelial barrier dysfunction and inflammatory responses ex vivo, which might be due to the interference with TLR5 signaling. The suppression of infection-induced inflammation by FOS may contribute to alleviating respiratory diseases in animals and humans.

## Figures and Tables

**Table 1:** Ingredient and nutrient composition of the experimental calf milk replacers.

	Experimental Period	Growing Period
Ingredient	g/kg	
Lactose or fructo-oligosaccharides ^1^ Lactose	2.7 32.3	
Whey powder	527	560
Delactosed whey powder	52	93
Whey protein concentrate	50	
Soy protein concentrate	60	55
Soluble wheat protein	50	41
Pea fiber	3	
Extruded wheat flour		41
Fat		
Lard	43	48
Tallow	68	77
Coconut oil	54	34
Lecithin	7.2	5
Emulsifier	7.2	5
Premix	10 ^2^	10 ^3^
Calcium formate	9.7	10
Citric acid	2.0	2
Sodium bicarbonate	4.0	4
Mono ammonium phosphate	3.5	2
Lysine	9.8	8
Methionine	2.4	3
Threonine	1.3	2
Aroma ^4^	0.2	
Nutrient	g/kg of Dry Matter unless Noted	
Dry matter, g/kg	975	976
Crude ash	82	74
Crude protein, N × 6.25	231	191
Crude fat	193	184
Lactose ^5^	453	455
Iron, ppm in dry matter	48	10

^1^ In experimental period, calves received either lactose (control) or 2.7 g/kg lactose was replaced with 92% pure FOS according to their allocated treatment. ^2^ The premix in experimental period provided (per kg of experimental diet) CP, 0.7 g; crude fat, 0.2 g; starch, 5.1 g; crude ash, 1.5 g; calcium, 16.6 mg; phosphorus, 7.6 mg; sodium, 0.7 mg; potassium, 7.3 mg; chloride, 13.1 mg; magnesium, 0.5 g; iron, 37.2 mg; copper, 7.4 mg; zinc, 109 mg; manganese, 43 mg; selenium, 0.3 mg; iodide, 6.9 mg; sulfur, 84 mg; vitamin A, 25,011 IU; vitamin D3, 4002 IU; vitamin E, 142 IU; vitamin C, 0.3 g; vitamin K3, 2.1 mg; vitamin B1, 8.2 mg; vitamin B2, 10.2 mg; vitamin B3, 34.9 mg; vitamin B5, 18.0 mg; vitamin B6, 9.4 mg; vitamin B12, 0.1 mg; biotin, 0.1 mg; choline, 0.4 g; and folic acid, 0.7 mg. ^3^ The premix in growing period provided (per kg of experimental diet) CP, 0.8 g; crude fat, 0.1 g; starch, 5.5 g; crude ash, 1.3 g; calcium, 17.0 mg; phosphorus, 8.1 mg; sodium, 0.8 mg; potassium, 7.8 mg; chloride, 13.2 mg; magnesium, 0.5 g; iron, 0.3 mg; copper, 4.0 mg; zinc, 100 mg; manganese, 43 mg; selenium, 0.3 mg; iodide, 1.0 mg; sulfur, 78 mg; vitamin A, 25,000 IU; vitamin D3, 4000 IU; vitamin E, 100 IU; vitamin C, 0.1 g; vitamin K3, 2.1 mg; vitamin B1, 8.2 mg; vitamin B2, 10.2 mg; vitamin B3, 34.8 mg; vitamin B5, 18.0 mg; vitamin B6, 6.3 mg; vitamin B12, 0.1 mg; biotin, 0.1 mg; choline, 0.4 g; and folic acid, 0.7 mg. ^4^ Aroma was purchased from LUCTAROM L, Lucta, Barcelona, Spain. ^5^ Calculated contents of control MR.

## Data Availability

The data presented in this study are available on request from the corresponding author.

## Supplementary Materials

The following are available online at https://www.mdpi.com/article/10.3390/nu13103514/s1, Figure S1: FOS have no effects on total cell numbers in BALF of calves, Figure S2: Effect of FOS on MHS-induced inflammation in PBECs, Figure S3: Anti-inflammatory effects of FOS in LPS- or flagellin-treated human airway epithelial cells, Table S1: The number of calves per treatment and parameter, Table S2: The number of calves positive for *M. haemolytica* in BALF over time.
