# Supplementary material for: Anti-Inflammatory Properties of Fructo-Oligosaccharides in a Calf Lung Infection Model and in Mannheimia haemolytica-Infected Airway Epithelial Cells
Source: Nutrients. 2021 Oct 6;13(10):3514. doi: 10.3390/nu13103514 (PMC8537102; doi:10.3390/nu13103514)
Supplement: Supplementary file 1 [file nutrients-13-03514-s001.zip › nutrients-1361062-supplementary.pdf]

Anti-inflammatory properties of fructo-oligosaccharides in a calf lung infection model and in *Mannheimia haemolytica*-infected airway epithelial cells

Yang Cai *et al*.

Table S1. The number of calves per treatment and parameter.

Parameters	Treatment	
	Control or FOS[1]	
	n per treatment	Collection frequency
Clinical scores	50	Weekly[2]
Blood collection[3]		bi-weekly
Week 0	50	
Week 2	20	
Week 4	20	
Week 6	20	
BALF collection[4]		bi-weekly
Week 1	20	
Week 3	20	
Week 5	20	
Week 7	20	
Lung scores	36 (Control)<br>39 (FOS)[5]	week 27

[1] One hundred male Holstein-Friesian calves 18d of age (43.2 ± 0.33 kg, means ± SEM) of German origin were used and assigned randomly to 2 treatments (n=50 calves/treatment).

[2] Weekly from week 1 to week 8.

[3] Blood samples were collected in all calves at arrival before the first MR feeding (week 0), and then were collected repeatedly in the subset of calves at week 2, 4 and 6.

[4] BALF samples were collected repeatedly for the same subset of calves at week 1, 3, 5 and 7.

[5] All remaining calves were scored at slaughter. In week 8, 10 calves per treatment were dissected for evaluating the degradation kinetics of FOS along the intestinal tract (not included in this paper). Four control calves and one FOS calf were excluded due to the death before week 27.

BALF, bronchoalveolar lavage fluid; FOS, fructo-oligosaccharides.

Table S2. The number of calves positive for *M. haemolytica* in BALF over time.

n=20 calves/treatment	Treatment		*P-value*	
	Control	FOS	Time[1]	Treatment[2]
Week 1	0	0	—	—
Week 3	4	4	0.11	>0.99
Week 5	16	14	<0.001	0.72
Week 7	16	15	<0.001	>0.99

[1] *P*-values apply to the time effect, different from week 1 control treatment.

[2] *P*-values apply to the treatment effect.

BALF, bronchoalveolar lavage fluid; FOS, fructo-oligosaccharides.

Supplementary Figures

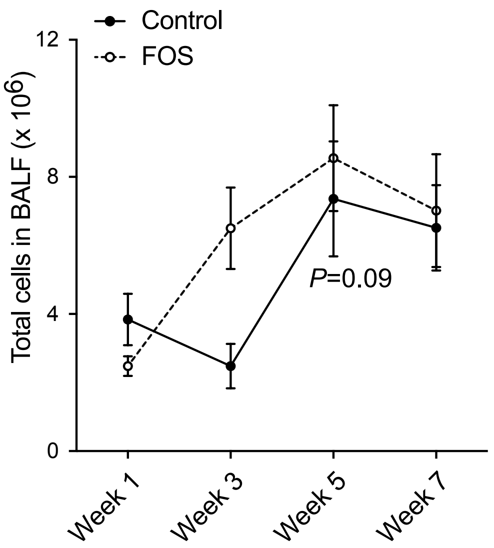

Figure S1. FOS have no effects on total cell numbers in BALF of calves. Number of total cells in BALF was determined for a subset of calves (n=40, 20 calves/treatment). Data are presented as means ± SEM. BALF, bronchoalveolar lavage fluid; FOS, fructo-oligosaccharides.

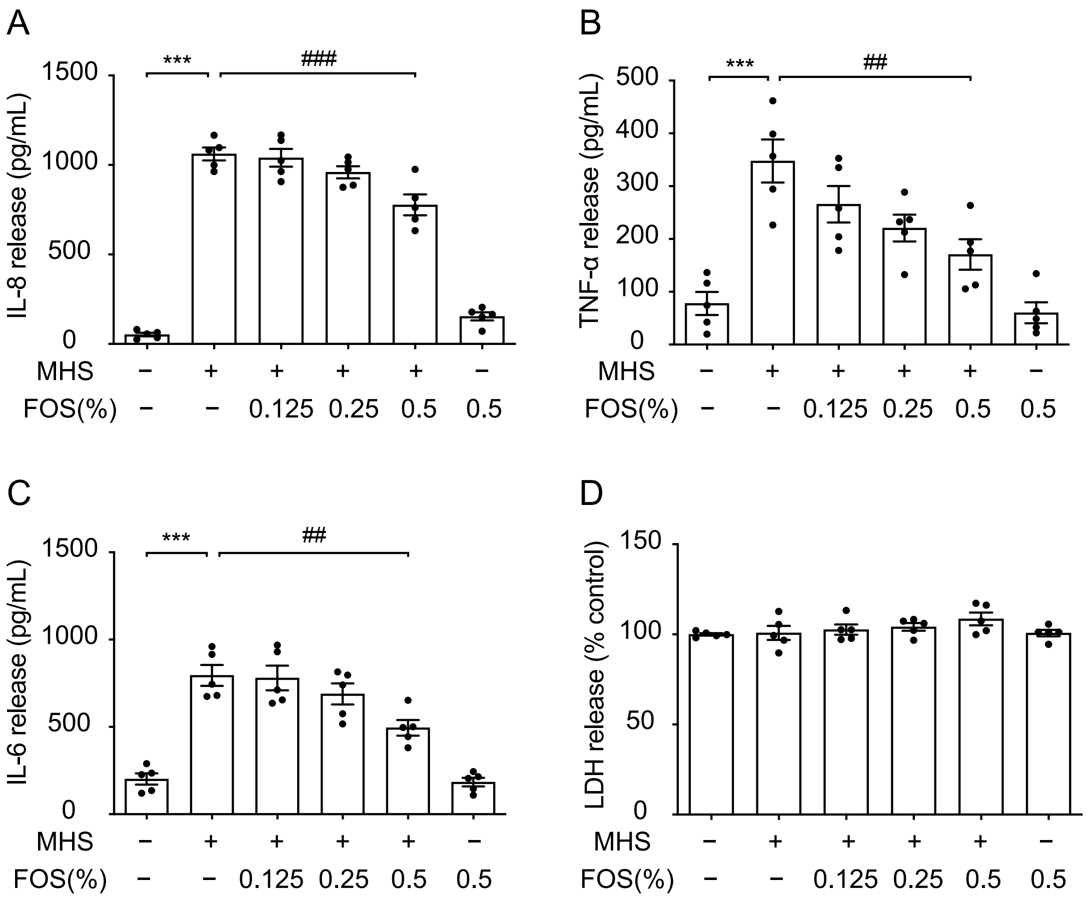

Figure S2. Effect of FOS on MHS-induced inflammation in PBECs. (A) IL-8, (B) TNF-α, (C) IL-6, and (D) LDH release was assessed in the supernatants of PBECs. Significantly different from control: ****P*<0.001. Significantly different from MHS: ##*P*<0.01; ###*P*<0.001. Data are presented as means ± SEM. All data shown are representative of five independent experiments (n=5 donor calves, one donor calf per experiment). FOS, fructo-oligosaccharides; IL, interleukin; LDH, lactate dehydrogenase; MHS, *M. haemolytica*-cultured supernatant; TNF, tumor necrosis factor.

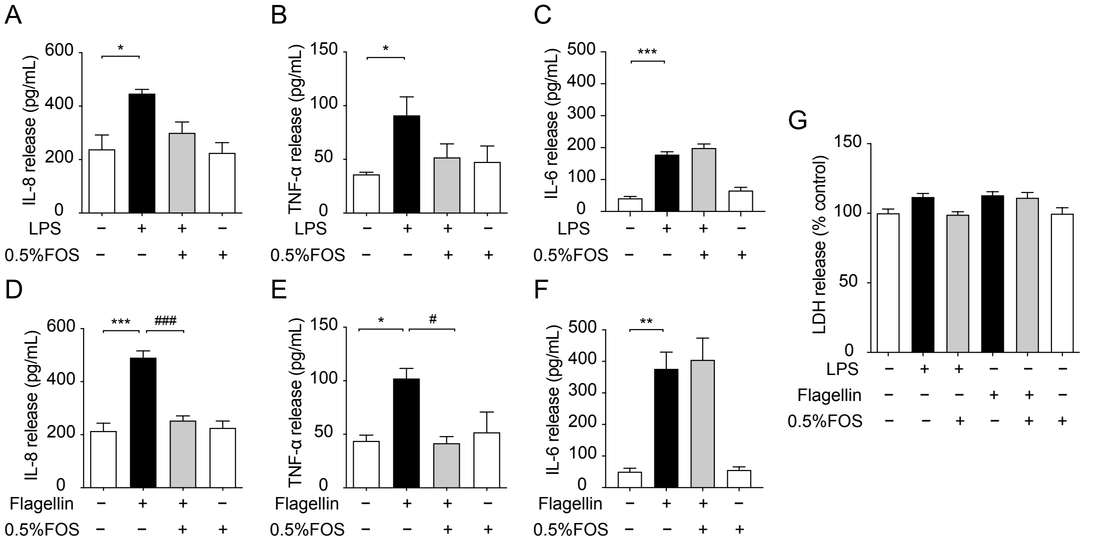

Figure S3. Anti-inflammatory effects of FOS in LPS- or flagellin-treated human airway epithelial cells. (A and D) IL-8, (B and E) TNF-α, (C and F) IL-6, and (G) LDH release were determined in the supernatants of LPS- or flagellin-treated human airway epithelial (A549) cells. Significantly different from control: **P*<0.05; ***P*<0.01; ****P*<0.001. Significantly different from flagellin: #*P*<0.05; ###*P*<0.001. Data are presented as means ± SEM. All data shown are representative of four independent experiments (n=4 cell generations, one generation per experiment). FOS, fructo-oligosaccharides; IL, interleukin; LDH, lactate dehydrogenase; LPS, lipopolysaccharides; TNF, tumor necrosis factor.
